# Electroencephalography Longitudinal Markers of Central Neuropathic Pain Intensity in Spinal Cord Injury: A Home-Based Pilot Study

**DOI:** 10.3390/biomedicines12122751

**Published:** 2024-11-30

**Authors:** Rab Nawaz, Ho Suen, Rahmat Ullah, Mariel Purcell, Shannon Diggin, Euan McCaughey, Aleksandra Vuckovic

**Affiliations:** 1 School of Computer Science and Electronic Engineering (CSEE), University of Essex, Colchester CO4 3SQ, UK; rab.nawaz@essex.ac.uk (R.N.);; 2 Department of Biomedical Engineering, James Watt School of Engineering, University of Glasgow, Glasgow G12 8QQ, UK; 3 Queen Elizabeth National Spinal Injuries Unit, Queen Elizabeth University Hospital, Glasgow G51 4TF, UK; 4 Cumulus Neuroscience Ltd., Belfast BT3 9DT, UK

**Keywords:** electroencephalography, neuropathic pain, spinal cord injury, correlation analysis, eyes open, eyes closed

## Abstract

Background: It is well known from cross-sectional studies that pain intensity affects brain activity as measured by electroencephalography (EEG) in people with neuropathic pain (NP). However, quantitative characterisation is scarce. Methods: In this longitudinal study, ten people with spinal cord injury-related NP recorded their home EEG activity ten days before and after taking medications over a period of several weeks. Results: The reduction in pain due to medications was accompanied by changes in the resting state EEG and its reactivity to eyes opening (EO) and closing (EC). There was a significant positive correlation between the frontal theta band and the intensity of pain (visual numerical scale) pre-medication (*p* = 0.007, Pearson R = 0.29) and theta, alpha, and lower beta (6–15 Hz) band power and the intensity of pain after post-medication over the frontal, central, and parietal cortices. Reactivity had a negative correlation with pain intensity at all locations and frequency bands and showed similar behaviour in wider frequency bands like 8–15 Hz at the occipital cortex and 2–12 Hz at the frontal cortex. Conclusions: EEG could be used to detect the intensity of NP to serve as a surrogate or pharmacodynamic marker.

## 1. Introduction

Neuropathic pain (NP) is a chronic debilitating condition caused by a lesion or a disease of the somatosensory system [1]. It affects between 7% and 10% of the general population [2] and around 50% of individuals with spinal cord injury (SCI) [3]. People with SCI often report shooting, burning, stinging, or stabbing pain at or below the level of injury despite having limited or even no real sensation. In spite of the availability of various pharmacological and non-pharmacological treatments, neuropathic pain remains refractory for many patients, seriously affecting their quality of life [4,5,6] and necessitating the exploration of novel diagnostic and therapeutic approaches.

Neuropathic pain causes functional and structural changes in the brain [7,8,9,10]. While functional magnetic resonance imaging provides the most accurate measurement, due to its size and price, it is not affordable for wider use. Electroencephalography (EEG) provides a portable and affordable option to monitor brain activity, offering insights into the neural mechanisms underlying chronic pain conditions. Previous studies of our and other research groups indicated that people with central NP due to SCI have distinguishable EEG features as compared to both able-bodied and people with SCI and no pain, and that these differences are evident in both resting and induced states during motor imagination [11,12]. A systematic review by Mussigmann et al. [13] highlighted that chronic neuropathic pain in general, including diverse patient groups, is characterised by increased theta (4–7 Hz) and high-beta (20–30 Hz) power, alongside decreased high-alpha and low-beta (10–20 Hz) power and reduced dominant alpha frequency, indicating altered thalamocortical dynamics [14]. Similarly, the meta-analysis of EEG studies of chronic pain in general found that increased resting state theta and beta power [15] is associated with pain.

In our previous study, we explored EEG features beyond the resting state power and showed that people with SCI-related NP have reduced reactivity, i.e., difference in EEG power between eyes opened (EO) and eyes closed (EC) state [11]. It is well known that alpha activity is dominant in neurologically healthy individuals during the eyes closed resting state [16]. Alpha desynchronises and consequently its amplitude drops upon opening eyes, due to visual stimulation. It is believed that this could be at least partially explained by cortical and thalamo-cortical information processing. Alpha desynchronisation is strongest in the occipital area but is generally widespread. Barry et al. [17] showed that localised desynchronisation also occurs in other frequency bands, frontal in delta, front central in theta, and posterior in the beta frequency range.

Tran et al. [6] reported reduced EO/EC EEG broadband (1–40 Hz) reactivity in people with NP due to SCI, as compared to able-bodied people as well as people with SCI without pain. Our research group found that this reduction could be mainly attributed to reduced alpha/theta desynchronisation in the EO state rather than to alpha synchronisation in EC state [12]. We further showed that this effect is present in subacute SCI [18], and that it can even be used as an EEG feature for machine learning, to identify people who are at risk of developing pain [19]. While the exact cause of reduced EO/EC reactivity is not well documented, it is probably related to altered multisensory integration in people with SCI [20] due to sensory and motor loss that could further be affected by thalamocortical dysrhythmia.

Several studies characterised EEG with respect to NP pain intensity, with somewhat contradictory results. Pain intensity scores positively correlated with increased EEG power in most bands, i.e., theta [21], alpha [22,23], whole beta [21,22], or gamma [24] bands, while negative correlations were also found in the alpha [25] and low-beta [25,26] band. Most of these studies were cross-sectional, but only Sarnthein et al. [27] and DiPietro et al. [22] compared how changes in pain intensity correlate with EEG one year after thalamotomy. Both studies reported the “normalisation” of EEG activity towards the EEG of pain-free people, but only in patients who reported a substantial (≥50%) reduction in pain. While the reduction in pain was immediate, it took at least 6 months for EEG changes to take place.

While a shift in dominant frequency is a well-documented phenomenon in NP [6,11,12,13,27], most studies analysed standard frequency bands, which might obscure findings. For example, if the dominant alpha peak is shifted towards 8 Hz, then calculating power in a frequency range of 8–12 Hz, assuming that the dominant peak is located at 10 Hz, effectively means missing half of the power of the real patient alpha band. In this study, we address this by looking at conventional and NP-modified frequency ranges.

Another notable gap in the literature is the lack of longitudinal studies with repeated measures to enable a correlation analysis between the daily variation of NP pain intensity and brain activity. Identifying this correlation is important to predict the effectiveness of pharmacological therapy for NP, which typically takes a few weeks to achieve its full potential, during which time multiple side effects might tempt patients to abandon the therapy. EEG might also be used as a supplement biomarker to complement a highly subjective widely used, visual numerical scale.

The correlation of EEG with reduced pain due to different treatments has been investigated in a handful of studies of other types of chronic pain. For example, Hunter et al. found a significantly positive correlation between theta cordance and pain reduction in fibromyalgia patients taking duloxetine for 12 weeks [28]. Levit and Saab [29] showed a positive correlation between the pain intensity and power in theta and beta bands, as well as theta/beta ratio in five migraine patients over 5 days that included periods pre- and post-severe migraine attack (treatment not specified).

In this study, we conducted a longitudinal analysis of EEG recordings in ten people with central NP due to SCI, recorded 10 days before and 10 days after taking medications over a period of several weeks. The importance of a longitudinal approach cannot be overstated. Chronic pain conditions are dynamic, with symptoms and brain activity fluctuating over time, even if recorded at the same time of the day. By examining EEG patterns across multiple sessions, we may identify consistent alterations in pre- and post-medication brain activity associated with day-to-day changes in pain intensity and short-term changes due to medication. We focus on resting state EEG and explore EO/EC reactivity as a candidate marker of NP. By bridging the gap between cross-sectional findings and longitudinal observations, we aim to contribute to the development of more effective pharmacodynamic and surrogate markers leading to targeted treatments for managing neuropathic pain.

## 2. Materials and Methods

### 2.1. Participants

Participants of both sexes aged between 18 and 70, with normal or corrected to normal vision, able to understand the task and to provide informed consent, were included in the study. Further inclusion criteria were the presence of chronic SCI, completeness A–D, injury level C3-T12, with moderate or high NP under the level of injury (intensity of 4 or higher on visual numerical scale), treated pharmacologically for at least 6 months. Exclusion criteria were the presence of any other type of pain larger than 3 on VNS, any other neurological condition known to affect EEG, expected changes in pain treatment throughout the study, computer illiteracy, no Internet access, the presence of severe concurrent medical conditions that would prevent participation in study procedures, or any contraindications to EEG recordings (skull irritation or infection).

The study included data from 10 SCI patients (including 4 females, age 61 ± 6 years, mean ± std). The study was approved by the London–Hampstead Research Ethics Committee, approval code: 22/PR/1090, dated 25 October 2022. Each participant signed the informed consent form, and the study was carried out in accordance with the Declaration of Helsinki [30].

Demographic information about the patients is shown in Table 1, reported in accordance with [31]. Note that sex is not reported for individual participants due to a high general prevalence of males being affected by SCI to avoid the incidental identification of female participants [32,33,34]. Seven participants were tetraplegic (injury level C) and three were paraplegic. Three patients had complete sensory and motor paralysis (ASIA A) [35], while others had incomplete injury having partially preserved sensation only (ASIA B) or partially preserved both the sensation and motor function (ASIA C and D). During the initial assessment session, participants filled out a brief pain inventory short form [36], providing information about the average pain (visual numerical scale (VNS), where 0 = pain, 10 = worst pain imaginable) and medications. Participants were taking first-line neuropathic pain medication [37], tricyclic antidepressants (Amitriptyline), and anticonvulsants (Pregabalin, Gabapentin), whilst some were taking muscle relaxants (Baclofen) and morphine-based drugs (Codeine) alongside over counter medications for mild pain (Paracetamol) (Table 1).

**Figure 1:**
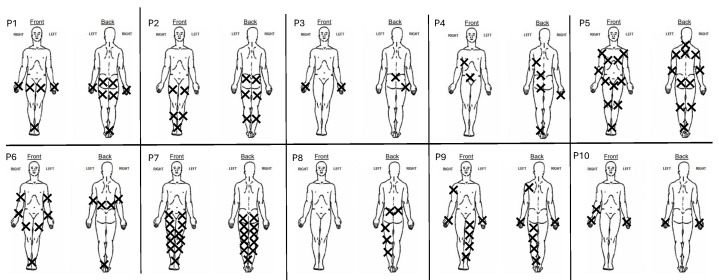
Location of most intense pain for each participant.

They also filled out a neuropathic pain symptoms Inventory [38] and the McGill questionnaire short form [39] providing information about the pain descriptors (Table 1). The location of the pain is shown on pain charts in Figure 1. All patients recorded EEG at the same time of the day, in the morning, immediately before taking medication and between 2 and 3 h post-medication. The system did not allow for any second EEG recording outside theses time limits.

### 2.2. Measures

#### 2.2.1. Pain Intensity

Pain intensity experienced just before the EEG recordings during each session was assessed by asking the participants to rate the intensity of pain using a VNS.

#### 2.2.2. EEG Recording

EEG recordings were obtained at home by participants after receiving one in person training session by a member of the research team. In case of participants being tetraplegic, a professional caregiver or a member of the family was trained to setup EEG while participants were trained to complete the tasks. The whole session lasted about 30 min and included recordings of the resting state EEG, auditory mismatched negativity task, and a 2 back visual memory task, but only the results based on the EEG resting state are presented herein.

At the end of each session, recorded data were automatically transferred to Cumulus Neuroscience Cloud for quality inspection. In the case of a poor-quality EEG recording, a member of the research team contacted the participant to provide additional online training. Daily recordings with only one experimental session were discarded and participants were asked to repeat the daily recording.

EEG was recorded using a 16-channel wearable wireless device with dry electrodes, manufactured by Cumulus Neuroscience, UK. The electrodes were placed according to the 10/10 international standard [40] on FT7, AF7, FPz, AF8, FT8, Fz, FCC3, Fz, FCC4, Cz, CPz, P3, Pz, P4, O1, and O2 locations. The headset featured flexible Ag/AgCl coated polymer sensors with a comb design to ensure stable and dermatologically safe contact with the scalp. The left mastoid served as the reference point, while the right mastoid was used for driven-bias, using single-use, snap-on electrodes attached to wires extending from the headset. The device has a medical CE mark and operates at a sampling rate of 250 Hz. The electronics and sensors were mounted on a flexible neoprene net for comfort, and the stretchable structure was designed to facilitate consistent placement by non-experts. An onboard processor and Bluetooth module transmitted EEG data to an Android tablet, from which it was then transferred to a secure cloud server for storage and processing.

During the resting state EEG recording, participants were asked to stay in a relaxed and wakeful state, without any particular task except for looking at the screen in the eyes open condition. Each resting state recording session lasted for 4 min, with the first 2 min recorded with eyes open and the last 2 min with eyes closed.

### 2.3. EEG Pre-Processing

Both EO and EC segments then underwent thorough inspection for artefact removal, incorporating both manual and automatic methods. Specifically, the artefact subspace reconstruction (ASR) [41] method within EEGLAB was employed to remove flat channels and reject transient artefacts. For channels that were excessively corrupted with noise or found to be flat, spherical spline interpolation [42] was applied to reconstruct them [43]. Additionally, muscular (EMG) and ocular (EOG) artefacts were removed by visually inspecting the time series and topographical maps generated by independent component analysis. All subsequent analysis was performed by grouping electrodes into four regions: frontal (Fz, AF7, Fpz, AF8), central (Cz, FCC3, FCz, FCC4), pariental (Pz, P3, CPz, P4), and occipital (O1, O2).

### 2.4. Power Spectrum Density

The cleaned EEG signal was imported into MNE-Python for further analysis. At least 60 s of the cleaned data were selected for analysis. These were divided into 2 s long signals. A 2-s epoch length was chosen to balance the stationarity of the signals [44]. All analyses are based on these epochs. The power spectral density (PSD) was computed using the ‘compute_psd()’ function in MNE-Python (mne-1.8.0-py3-none-any), employing Welch’s method with a frequency range set between 1 Hz and 40 Hz. This function was applied to the EEG epochs to effectively estimate the PSD within the specified frequency band, enabling a detailed analysis of the oscillatory activity in the EEG signals.

To analyse the PSD data, two metrics were computed for all frequency bands of interest: absolute band power and relative band power. The absolute power for each frequency band was calculated using the trapezoidal integration method in Python, as shown in Equation (1):

(1) $$\mathrm{absolute}\_\mathrm{band}\_\mathrm{power}=\int_{f_{\mathrm{low}}}^{f_{\mathrm{high}}}\mathrm{psd}\left(f\right)\;df$$

where $f_{\mathrm{low}}$ and $f_{\mathrm{high}}$ are cut off frequencies of high- and low-pass filters, respectively. The relative band power was determined by first computing the absolute power for the specific band, and then dividing it by the total power across all frequencies. The total power was obtained by integrating the entire PSD spectrum, as shown in Equation (2):

(2) total_power=∫psd(f)df

The relative power was then calculated using Equation (3):

(3) $$\mathrm{relative}\_\mathrm{band}\_\mathrm{power}=\frac{\mathrm{band}\_\mathrm{power}}{\mathrm{total}\_\mathrm{power}}$$

This normalisation to total power ensured that the relative power reflected the proportion of power within the specified frequency band relative to the overall EEG power spectrum.

### 2.5. Statistical Analysis

The data for this study consisted of multiple observations over multiple time points. As we could not assume normal distribution, a Wilcoxon signed-rank test [45,46] was used for all statistical analyses.

For each participant, the Wilcoxon signed-rank test was applied separately to compare the band power values between the two conditions (pre-medication vs. post-medication) for each session. Due to the multiple comparisons inherent in our analysis, we addressed the risk of Type I error inflation through the use of Fisher’s method for combining *p*-values [47].

The combined chi-square statistic and the resulting *p*-value enable a holistic assessment of the overall effect across all participants, enhancing the robustness and interpretability of the findings. Statistical analyses were performed using Python, leveraging libraries such as SciPy for the Wilcoxon signed-rank tests and Statsmodels for the combination of *p*-values (*p* < 0.05) using Fisher’s method [48,49].

### 2.6. Linear Regression Analysis

To examine the relationship between the self-reported pain levels and the changes in EEG power across different frequency bands, we performed a linear regression analysis using a robust approach to account for potential outliers. A Pearson coefficient (R, p) was calculated between EEG parameters and pain intensity.

Initially, a linear regression was conducted to identify potential outliers in the data. This involved computing the regression slope, intercept, correlation coefficient (*r*-value), *p*-value, and standard error using the linregress function from the SciPy library [49]. The residuals were then calculated as the difference between the observed power differences and the predicted values from the initial regression. Outliers were identified as data points where the absolute residuals exceeded 2.5 standard deviations from the mean residual [50].

Subsequently, the data were filtered to exclude these outliers, resulting in a subset of inlier data points. A final linear regression was then performed on these inlier data to obtain the final regression parameters, including the slope, intercept, *r*-value, *p*-value, and standard error. This two-step approach ensured that the regression analysis was not unduly influenced by extreme values, thus providing a more reliable estimation of the relationship between pain levels and EEG [51]. A Holm–Bonferroni correction for multiple comparison was applied.

For the regression analysis, EEG was analysed in conventional theta (4–8 Hz), alpha (8–12 Hz), and beta (12–30 Hz) bands; there was a shift towards lower frequencies in the following frequency bands: theta (4–8 Hz), delta/theta (2–6 Hz), alpha (8–12 Hz), theta/alpha (6–10 Hz), beta (13–30 Hz), high alpha/low beta (10–15 Hz), and high beta (20–30 Hz). Analysis was performed for both absolute and relative power due to changes in EEG power as a result of taking medication.

## 3. Results

The analysis of the resting EEG state including EO/EC reactivity and the influence of medications on EO and EC resting EEG are presented first. This is followed by three correlation analyses (Pearson correlation coefficient, *p* < 0.05) between EEG-based markers and pain.

### 3.1. EEG Power in Eyes Opened vs. Eyes Closed Resting State Before and After Medications

**Figure 2:**
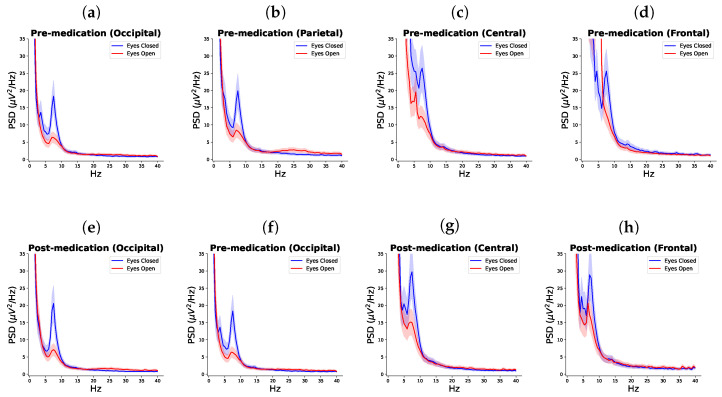
Comparison of PSD in eyes open and eyes closed states within each brain lobe for pre-medication session (**a**–**d**) and post-medication session (**e**–**h**).

Difference between eyes opened and eyes closed conditions were significant in all frequency bands and at all cortical locations (frontal, central, parietal, and occipital) except for pre-medication beta (*p* = 0.1298) at the frontal cortex (Table 2, Figure 2).

A difference between the EC before vs. post-medication was statistically significant in the theta band in the frontal cortex (*p* = 0.047, $\chi^{2}$ = 29.086), in the alpha band in the parietal cortex (*p* = 0.047, $\chi^{2}$ = 29.108), and in the beta band in the occipital cortex (*p* = 0.043, $\chi^{2}$ = 29.477).

**Figure 3:**
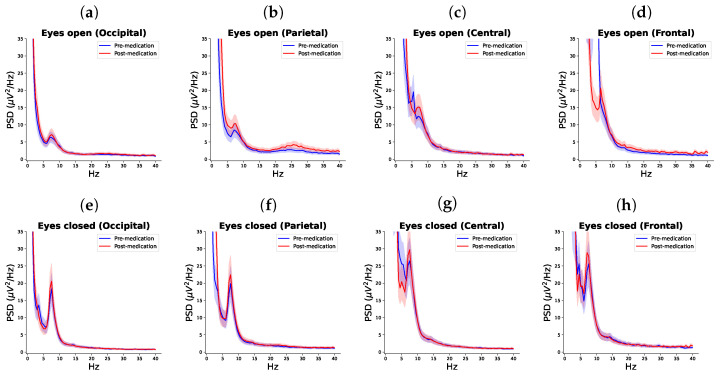
Comparison of PSD in pre-medication and post-medication sessions within each brain lobe in eyes open state (**a**–**d**) and eyes closed state (**e**–**h**).

A difference in the EO before vs. post-medication was statistically significant in the theta band at the parietal and the occipital cortices (*p* = 0.036, $\chi^{2}$ = 29.108 and *p* = 0.031, $\chi^{2}$ = 30.744, respectively). In the alpha band, the difference was statistically significant at the central region (*p* = 0.046, $\chi^{2}$ = 29.182) and in the beta band there was no statistically significant difference before EC before and after taking medications (Table 3, Figure 3). In all cases, apart from the frontal EO theta, EEG power increased post-medication.

### 3.2. Correlation Between Relative EEG Power and Pain Intensity

In the eyes-open relaxed state, before taking medications, there was a statistically significant positive correlation between the theta band power and pain intensity on the VNS at the frontal cortex (*p* = 0.009, R = 0.29, respectively). This means that more theta corresponded to a higher intensity of pain.

After taking medications, there was a positive statistically significant correlation between EEG power and pain intensity between theta/alpha band at the central and frontal regions (*p* = 0.001, R = 0.37 and *p* = 0.016, R = 0.26, respectively), at the alpha band in four regions (frontal *p* = 0.001, R = 0.36, central *p* = 0.0001, R = 0.38, parietal *p* = 0.019, R = 0.26 and occipital *p* = 0.027, R = 0.24), and in the alpha/beta band at all four regions (frontal *p* = 0.001, R = 0.36, central *p* = 0.0003, R = 0.38, parietal *p* = 0.003, R = 0.31, occipital *p* = 0.008, R = 0.29). Given the proximity and overlap between these frequency bands, they most likely present one frequency band.

A negative correlation was found with the delta/theta band in the central region (*p* = 0.033, R = −0.23), and with the higher beta band at the occipital cortex (*p* = 0.016, R = −0.26 and *p* = 0.05).

**Figure 4:**
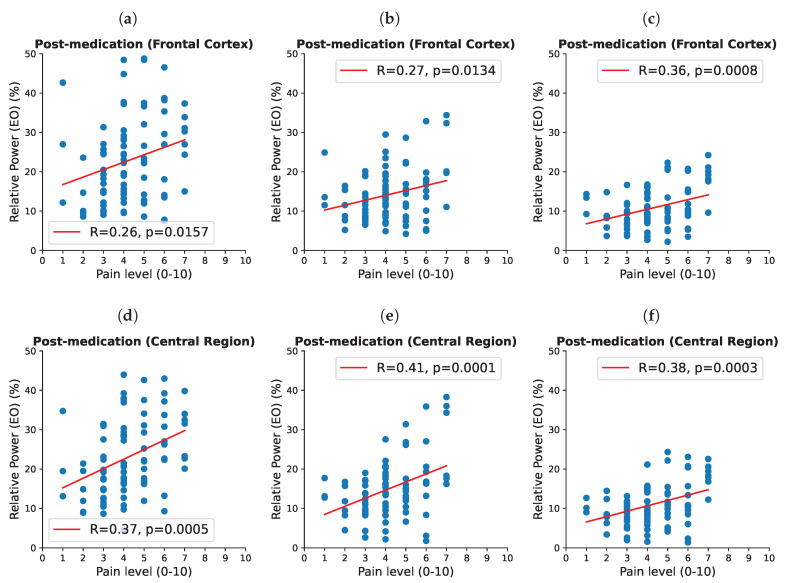
Correlation (Pearson coefficient) between EEG power after taking medications in the frontal cortex for the theta/alpha (6–10 Hz), alpha (8–12 Hz), and alpha/beta (10–15 Hz) (**a**–**c**), respectively, and in the central region for the theta/alpha (6–10 Hz), alpha (8–12 Hz) and alpha/beta (10–15 Hz) (**d**–**f**), respectively. R and *p* values are shown in figures.

Figure 4 shows a correlation between EEG power post-medication at the frontal and central regions for different frequency bands. In the eyes-closed resting state, there was no statistically significant correlation (*p* < 0.05). Detailed results for each frequency band and each cortical area are presented in the Supplementary Materials.

### 3.3. Correlation Between the Relative Changes in EEG and Relative Changes in Pain

Changes in EEG power and pain intensity post-medication were calculated with respect to the corresponding values pre-medication using the formula (post–pre)/pre. In this way, both EEG power and pain intensity were normalised.

When either absolute or relative EEG power was implemented in the formula above, there was no significant correlation between EEG and pain intensity in any frequency band, even after correction for multiple comparisons. Detailed results for each frequency band and each cortical area are presented in the Supplementary Materials.

### 3.4. Correlation Between the EO/EC Ratio (Reactivity) and Pain Intensity

After corrections for multiple comparisons, there were no statistically significant correlations. However, upon a closer look, neighbouring/overlapping frequency bands consistently showed a significant correlation at the same cortical location, indicating a frequency cluster of activity that might be subject to false negative rate outcome. For instance, for the absolute EEG power, pre-medication, a statistically significant negative correlation between EO/EC ratio and pain intensity (VNS) was found at the occipital cortex in the alpha and alpha/beta EEG power (*p* = 0.024, R = −0.24 and *p* = 0.041, R = −0.24, respectively), which, given the frequency and spatial overlap, most likely present one frequency band.

After taking medications, a statistically significant negative correlation was found at the frontal cortex between the delta/theta, delta, and theta/alpha EEG power and pain intensity (*p* = 0.041, R = −0.22; *p* = 0.039, R = −0.22; *p* = 0.028, R = −0.24, respectively). Again, given the frequency and spatial overlap, these most likely present one frequency band.

Taken together, these results indicate that the higher EO/EC ratio (either more EC or less EO) is related to the higher pain intensity, though different frequency bands show significant correlation in different cortical areas.

For the relative EEG post-medication, a significant positive correlation was found at the occipital cortex for the alpha and alpha/beta bands (*p* = 0.024, R = 0.25 and *p* = 0.034, R = 0.23, respectively), the two probably reflecting activity in the high alpha band. Detailed results for each frequency band and each cortical area are presented in the Supplementary Materials.

## 4. Discussion

This study investigated the longitudinal EEG band power changes in SCI patients with central neuropathic pain, focusing on absolute and relative power in the theta, alpha, and beta bands across different brain regions.

In our previous study [18], we showed that, in contrast to their able-bodied counterparts, people with SCI and NP did not have a statistically significant difference between EEG power in EO and EC state at all electrode locations. This is due to disrupted thalamocortical pathways that likely result in altered multisensory integration in people with SCI in general [19] that might be further aggravated by the presence of NP. However, in this study, probably because we averaged results across several electrodes over the same cortical region, statistically significant differences were found in each location except in the beta band frontally prior to taking medication.

Previous studies provided inconclusive results with respect to the correlation between the intensity of pain and EEG power [21,22,23,24,25,26]. In this study, we found that all statistically significant differences between pre- and post-medication EEG indicated increase in power post-medication, apart from frontal EO theta which decreased. A decrease in frontal theta and a general increase in alpha would be expected as they would counterbalance the general markers of NP, increased theta and decreased alpha power [13]. It is possible that an increase in EC theta band power over parietal and occipital cortices was due to medications. All participants took at least one of the first line medications for the treatment of NP (anticonvulsants Gabapentin, Pregabalin, and tricyclic antidepressant Amitriptyline); one took second line medication, morphine-based opioid Codeine; and four took the antispastic drug Baclofen. In addition, six took over-the-counter analgetic Paracetamol. The effect of anticonvulsants Gabapentin and Pregabalin on resting state EEG is well documented, showing a small albeit significant increase in theta band power and a reduction in the dominant alpha frequency in the parietal cortex [52,53,54,55]. The effect of the tricycling antidepressant was mostly assessed during sleep, but a resting-state EEG study showed that they increase delta and theta band power [56]. Codeine [56] and Baclofen [57] might also increase delta and theta power, while Paracetamol in therapeutic doses does not influence resting state EEG [56].

Medications affect EEG in two ways: through a general effect that can be detected in healthy people and indirectly by affecting a particular neurological condition. Most aforementioned studies tested the general effect on a healthy population. A common finding is that the medications used for the treatment of NP increase theta band power dominantly in the parietal region. This effect should be taken into account in future studies of EEG pharmacodynamic markers of NP in the patient population.

Many studies exploring the correlation between EEG and NP combined patients with an NP of different aetiologies, taking a variety of medications [21,26,27]. Out of four published studies on NP makers in chronic SCI, three recruited patients with different levels and completeness of injuries, taking different medications [11,23,58]. One study that included paraplegic patients only (injury to the lower spine) was from our group [12], and in that study, we showed that the presence of pain in lower limbs also affects the activity of the sensory-motor cortex of non-painful upper limbs, i.e., the presence of central neuropathic pain alters sensory activity globally. That and the notion that NP in SCI does not depend on the level or completeness of injury [3] further corroborate inclusion criteria in this study.

A challenge in testing the effect of drugs on people with SCI is that most patients take multiple NP drugs [37] and the only way to test a single medication would be to recruit patients who recently started NP treatment because medications are introduced one at the time. In that case, patients with different aetiologies of NP should be included, because SCI is a relatively small patient population. Pharmacodynamic markers based on EEG are relatively novel, and for testing the effect of new medications, EEG markers should be combined with mechanical or thermal markers to complement the findings.

There are multiple non-pharmacological methods to treat NP, and EEG could also be used to test the effectiveness of these methods on the reduction in pain. NICE recommends physical exercises, psychological therapy, and acupuncture [59]—although there are more methods based on using technology focusing on neuromodulation, directly targeting brain activity [60,61,62]. Some therapies such as neurofeedback [63], mental rotation [64], and motor imagery [65,66] require the voluntary modulation of brain activity and visual feedback, and as such, would be particularly well suited for assessment with EEG-based markers of NP. While longitudinal home-based studies are not suitable for high-density EEG recordings, further cross-sectional studies in a research setting would facilitate multichannel recordings to enable source localisation and connectivity analysis.

As reported previously, participants in this study had a lower dominant alpha frequency of around 8 Hz. For that reason, we performed a correlation analysis with several overlapping bands to account for a shift in the alpha band towards theta, and the difference between the lower and higher beta bands. This showed the clustering of activity in wider frequency ranges spanning theta/alpha/lower betta band or delta/theta/lower alpha band, which probably reflected the activity of common sources, shifting in frequency due to thalamo-cortical dysrhythmia.

For example, the most robust feature in the EO state, present in all cortical areas and that had a significant positive correlation with pain intensity (VNS) was a relative power in a wide frequency range theta/alpha/low beta (6–15 Hz), indicating that higher EEG power corresponds to higher pain intensity. This was, however, only noticeable after taking medications. Prior to taking medications, the frontal theta band had a positive correlation with the intensity of pain. This is in accordance with previous studies, showing increased levels of theta activity in the presence of NP [12,27].

Our results are in line with those of Jensen et al. [23], who reported increased theta and decreased alpha power in people with SCI and NP, but found contradictory results that more alpha power corresponds to more pain, at least post-medication. Post-medication results showed that more pain corresponds to less theta in the central region as opposed to positive correlation between frontal theta band power and pain intensity pre-medication.

It is of interest to note that there was no correlation in EC state, although most resting state studies analysed EEG in the eyes-closed state to reduce EOG and facial muscle artefacts [21,22,23], but Michels et al. [21] reported similar results in EO and EC states. It is possible that the effect in EC was small and would require a larger number of participants to be detected.

An obvious limitation of this and other similar studies is that a VNS was used to report the intensity of pain, although it is highly subjective and therefore might vary greatly between people. In this study, this issue is somewhat counterbalanced by repeated measures from the same participants. In addition, in order to minimise intersubject variability, we also calculated the relative change in EEG power and pain post-medication with respect to pre-medication. No significant correlation was found. The reason for this might be that, with only 10 participants, we did not have sufficient power to notice the effect, which even without correction for multiple comparison showed significant correlation in the central lower beta band only. Studies of different types of chronic pain showed that pain relief due to medications or interventions is accompanied by changes in EEG [28,29,31,32,33,34], though the direction of change was inconclusive and the regression analysis of longitudinal studies were missing.

Finally, we also used EO/EC reactivity as a marker of pain intensity. Similarly to the analysis of relative power, we noticed clustering over a wider frequency range in certain areas, with all correlations, irrespective of the frequency band, location, or time of measurement (pre- or post-medication) being negative, indicating that larger EC power (or in the case of SCI, more likely smaller EO power) is related to a higher intensity of pain. An interesting question would be to test whether reduced EO/EC reactivity also exists in people with central NP due to other neurological problems or in people with thalamo-cortical disrhytmia due to neurological conditions other than NP.

A noteworthy effect which repeated throughout the analysis of different features was that clusters of wider frequency ranges showed similar behaviour and might be reflecting the same source of activity. Unfortunately, we could not perform source analysis due to a small number of electrodes. This was a necessary compromise between functionality and ease of use, to allow participants to collect repeated measurements in the comfort of their homes. Although EEG recording in laboratory-controlled settings would undoubtedly provide better quality recordings, even from the larger number of electrodes, that would present too large a burden to participants. Any widespread affordable use of EEG-based prognostic or pharmacodynamic markers of any neurological condition would inevitably necessitate repeated EEG measurements over a prolonged time period and at patients’ homes, and thus, conducting a study with a small number of electrodes and simple experimental settings is necessary in order to mimic real-life situations. Future longitudinal studies on a larger number of participants should be able to reveal additional smaller effect sizes relationships between pain and EEG, which could not be detected in the study.

In this study, we found several feature candidates to characterise the intensity of pain. While this is a pilot study on a small number of participants, albeit with a much larger number of repeated sessions than reported in the literature, studies on a larger number of participants would increase the statistical power and enable multifactorial analysis, in order to identify the most robust feature and further test the relevance of EC/EO reactivity as a marker of pain. Further profiling might be possible based on different phenotypes of central NP in SCI [67] or on different neurological conditions leading to NP in other patient populations.

## 5. Conclusions

The main novelty of this pilot study is its patient self-managed longitudinal nature and the exploration of EO/EC reactivity as a candidate feature to characterise the intensity of pain. For the first time, patients were able to record EEG in the comfort of their homes and to safely upload their data for analysis. Demonstrating the feasibility of this approach even with highly disabled users opens the door to larger-scale studies of different neurological conditions.

The analysis of EEG showed that the reduction in pain due to medications was accompanied by a reduction in frontal theta, and an increase in widespread alpha changes in EO/EC reactivity. The most robust positive correlation between pain and relative EEG power was with a wide 6–15 Hz band over the whole cortex. The correlation between relative pre/post-medication changes in EEG and the intensity of pain remains inconclusive and would require a larger study. The correlation between the EO/EC reactivity and pain was negative and involved wider frequency bands, spanning theta, alpha, and low beta over the frontal and central areas. In future studies, a larger number of participants would allow one to apply machine learning methods, rather than correlation analysis, to establish a correlation between EEG features and pain intensity for individual patients.

Surrogate EEG markers would be helpful in complementing highly subjective pain ratings with VNS, or to assess the intensity of pain in nonverbal patients. Pharmacodynamic markers could provide the early prediction of the effectiveness of a therapy, and might even manifest before the onset of sensory signs of pain, in a similar way to how early markers of NP present before the onset of pain sensation [18,19].

In addition, EEG can be used as a response marker to assess the effectiveness of nonpharmacological treatments in NP, and would be particularly well suited for methods based on neuromodulation using technology that directly modulates EEG activity.

## Figures and Tables

**Table 1:** Participants’ demographic information. ASIA—American Spinal Injury Association Impairment Scale [35]. Information about average pain recall, pain descriptors, and medications taken during the initial assessment.

ID	Age Bin	ASIA/Level	Aver Pain (VNS)	Descriptor	Medications
P1	45–59	B/C6	7	Burning, shooting, tingling	Amitriptyline, paracetamol
P2	60–74	B/T6	6	Pulsing, sharp, gnawing, burning, stinging, freezing	Pregabalin, Gabapentin
P3	45–59	B/C5	5	Burning, freezing, tingling	Pregabalin, Amitriptyline, Paracetamol
P4	45–59	D/C3	5	Burning, pressing, stabbing, flashing	Pregabalin, Gabapentin
P5	45–59	A/C4	7	Shooting, throbbing, hot, stabbing, tingling, pressing	Amitriptyline, Codeine
P6	60–74	C/C5	6	Burning, pressing, stabbing	Amitriptyline, Pregabalin, Paracetamol
P7	60–74	A/T8	8	Pulsing, throbbing, stabbing, cramping, tingling	Pregabalin, Baclofen, Paracetamol
P8	60–74	A/L1,2	7	Shooting, throbbing, stabbing, sharp, hot, pulling, stinging, cramping	Amitriptyline, Pregabalin, Baclofen
P9	60–74	C/C4	6	Pulsing, stabbing, mulling, burning, tingling, pulling	Pregabalin, Baclofen, Paracetamol
P10	45–59	B/C3	5	Burning, pressure, shooting, stabbing	Gabapentin, Paracetamol, Baclofen

**Table 2:** Statistical significance (*p* < 0.05) of difference between EO and EC resting state EEG before (pre) and after (post) taking medications, over different areas of the cortex. $\chi^{2}$: Chi-square statistics, and related *p*-value (significance level *p* < 0.05).

			Frontal	Central	Parietal	Occipital
**Theta (4–8 Hz)**	Pre	*p*	$1.218\times 10^{-7}$	$6.441\times 10^{-6}$	$1.679\times 10^{-6}$	$3.250\times 10^{-7}$
		$\chi^{2}$	67.443	63.094	60.531	64.883
	Post	*p*	$5.851\times 10^{-5}$	$1.880\times 10^{-4}$	$3.623\times 10^{-6}$	$3.863\times 10^{-10}$
		$\chi^{2}$	50.725	47.355	58.458	80.650
**Alpha (8–12 Hz)**	Pre	*p*	$1.057\times 10^{-7}$	$6.955\times 10^{-10}$	$1.341\times 10^{-8}$	$1.464\times 10^{-7}$
		$\chi^{2}$	67.811	80.518	73.101	66.965
	Post	*p*	$9.196\times 10^{-6}$	$2.542\times 10^{-6}$	$1.513\times 10^{-6}$	$1.225\times 10^{-9}$
		$\chi^{2}$	55.914	59.416	60.809	79.112
**Beta (12–30 Hz)**	Pre	*p*	0.01298	0.01208	$1.998\times 10^{-2}$	$1.750\times 10^{-3}$
		$\chi^{2}$	224.826	34.147	32.368	40.553
	Post	*p*	$1.528\times 10^{-6}$	$8.570\times 10^{-4}$	$1.390\times 10^{-4}$	$2.560\times 10^{-4}$
		$\chi^{2}$	60.783	42.789	48.243	46.443

**Table 3:** Statistical significance of difference between EO before and after medication and EC before and after medications over different areas of the cortex. $\chi^{2}$: Chi-square statistics and related *p*-value (significance level *p* < 0.05).

			Frontal	Central	Parietal	Occipital
**Theta (4–8 Hz)**	EO	*p*	**0.047**	0.288	0.146	0.141
		$\chi^{2}$	**29.086**	20.829	24.297	24.439
	EC	*p*	0.099	0.581	**0.036**	**0.031**
		$\chi^{2}$	26.026	16.163	**30.179**	**30.744**
**Alpha (8–12 Hz)**	EO	*p*	0.145	0.446	**0.047**	0.209
		$\chi^{2}$	24.301	18.140	**29.108**	22.531
	EC	*p*	0.397	**0.046**	0.513	0.516
		$\chi^{2}$	18.921	**29.182**	17.144	17.098
**Beta (12–30 Hz)**	EO	*p*	0.510	0.790	0.075	**0.043**
		$\chi^{2}$	17.197	13.027	27.200	**29.477**
	EC	*p*	0.675	0.861	0.091	0.166
		$\chi^{2}$	14.811	11.727	26.386	23.685

## Data Availability

Data are available from the authors upon request.

## Supplementary Materials

The following supporting information can be downloaded at https://www.mdpi.com/article/10.3390/biomedicines12122751/s1, Table S1: Absolute Power difference of Pre-medication and Post-medication. (EO/EC); Table S2: Absolute Power difference of Eyes Close and Eyes Open. ((Pre—Post)/Pre); Table S3: Absolute Power difference of Eyes Close and Eyes Open. (Pre/Post); Table S4: Relative Power difference of Pre-medication and Post-medication. (EO/EC); Table S5: Relative Power difference of Eyes Close and Eyes Open. (Pre—Post)/Pre; Table S6: Relative Power difference of Eyes Close and Eyes Open. (Pre/Post); Table S7: Relative Power EC of Pre-medication and Post-medication; Table S8: Relative Power EO of Pre-medication and Post-medication.
