# Supplementary material for: Electroencephalography Longitudinal Markers of Central Neuropathic Pain Intensity in Spinal Cord Injury: A Home-Based Pilot Study
Source: Biomedicines. 2024 Nov 30;12(12):2751. doi: 10.3390/biomedicines12122751 (PMC11672874; doi:10.3390/biomedicines12122751)
Supplement: Supplementary file 1 [file biomedicines-12-02751-s001.zip › biomedicines-3313324 supplementary.pdf]

Table S1: Absolute Power difference of Pre-medication and Post-medication. (EO/EC)

Band	Lobs	R	P-value
**Pre-medication**			
2-6 (Theta)	Occipital	-0.01	0.905
	Parietal	-0.14	0.216
	Central	-0.06	0.556
	Frontal	0.05	0.674
4-8 (Theta)	Occipital	-0.06	0.618
	Parietal	-0.10	0.931
	Central	-0.04	0.695
	Frontal	-0.03	0.804
6-10 (Alpha)	Occipital	-0.17	0.112
	Parietal	-0.05	0.669
	Central	0.07	0.504
	Frontal	0.04	0.706
8-12 (Alpha)	Occipital	-0.24	0.024
	Parietal	-0.16	0.150
	Central	-0.01	0.894
	Frontal	-0.10	0.393
10-15 (Low Beta)	Occipital	-0.22	0.041
	Parietal	-0.16	0.139
	Central	-0.10	0.379
	Frontal	-0.07	0.517
20-30 (High Beta)	Occipital	-0.01	0.918
	Parietal	0.08	0.444
	Central	0.03	0.767
	Frontal	-0.05	0.652
12-30 (Beta)	Occipital	-0.05	0.670
	Parietal	0.01	0.925
	Central	0.00	0.983
	Frontal	-0.07	0.546
**Post-medication**			
2-6 (Theta)	Occipital	-0.04	0.732
	Parietal	-0.11	0.349
	Central	-0.17	0.129
	Frontal	-0.22	0.041
4-8 (Theta)	Occipital	-0.04	0.708
	Parietal	-0.09	0.400
	Central	-0.06	0.608
	Frontal	-0.22	0.039
6-10 (Alpha)	Occipital	0.14	0.203
	Parietal	0.00	0.995
	Central	-0.12	0.295
	Frontal	-0.24	0.028
8-12 (Alpha)	Occipital	0.14	0.193
	Parietal	0.09	0.438
	Central	-0.15	0.169
	Frontal	-0.21	0.056
10-15 (Low Beta)	Occipital	0.14	0.198
	Parietal	0.02	0.878
	Central	-0.12	0.295
	Frontal	-0.15	0.171
20-30 (High Beta)	Occipital	-0.02	0.856
	Parietal	-0.20	0.066
	Central	-0.22	0.048
	Frontal	-0.13	0.226
12-30 (Beta)	Occipital	-0.02	0.855
	Parietal	-0.14	0.211
	Central	-0.17	0.127
	Frontal	-0.14	0.193

Table S2: Absolute Power difference of Eyes Close and Eyes Open. ((Pre - Post) / Pre))

Band	Lobs	R	P-value
**Eyes Close**			
2-6 (Theta)	Occipital	0.01	0.952
	Parietal	0.13	0.230
	Central	0.12	0.267
	Frontal	-0.03	0.796
4-8 (Theta)	Occipital	-0.03	0.816
	Parietal	0.19	0.094
	Central	0.07	0.531
	Frontal	-0.13	0.246
6-10 (Alpha)	Occipital	0.08	0.458
	Parietal	0.18	0.091
	Central	0.00	0.976
	Frontal	0.03	0.773
8-12 (Alpha)	Occipital	0.03	0.760
	Parietal	0.16	0.140
	Central	0.13	0.240
	Frontal	0.18	0.096
10-15 (Low Beta)	Occipital	-0.08	0.457
	Parietal	0.05	0.626
	Central	0.11	0.318
	Frontal	0.07	0.553
20-30 (High Beta)	Occipital	0.01	0.894
	Parietal	0.03	0.760
	Central	0.01	0.916
	Frontal	0.02	0.885
12-30 (Beta)	Occipital	-0.01	0.949
	Parietal	-0.09	0.453
	Central	0.16	0.170
	Frontal	0.08	0.463
**Eyes Open**			
2-6 (Theta)	Occipital	-0.17	0.138
	Parietal	0.01	0.951
	Central	0.12	0.281
	Frontal	-0.02	0.846
4-8 (Theta)	Occipital	-0.04	0.743
	Parietal	-0.06	0.607
	Central	0.08	0.486
	Frontal	0.04	0.702
6-10 (Alpha)	Occipital	-0.19	0.081
	Parietal	-0.09	0.431
	Central	0.00	0.986
	Frontal	-0.15	0.173
8-12 (Alpha)	Occipital	-0.20	0.073
	Parietal	-0.10	0.368
	Central	-0.10	0.358
	Frontal	-0.02	0.835
10-15 (Low Beta)	Occipital	0.12	0.269
	Parietal	-0.06	0.592
	Central	-0.06	0.591
	Frontal	-0.05	0.670
20-30 (High Beta)	Occipital	0.11	0.337
	Parietal	-0.02	0.884
	Central	0.03	0.772
	Frontal	0.01	0.962
12-30 (Beta)	Occipital	-0.02	0.893
	Parietal	-0.07	0.520
	Central	0.06	0.568
	Frontal	-0.03	0.817

Table S3: Absolute Power difference of Eyes Close and Eyes Open. (Pre / Post)

Band	Lobs	R	P-value
	**Eyes Close**		
2-6 (Theta)	Occipital	0.03	0.801
	Parietal	0.20	0.070
	Central	0.16	0.147
	Frontal	0.19	0.079
4-8 (Theta)	Occipital	0.02	0.837
	Parietal	0.11	0.313
	Central	0.18	0.097
	Frontal	0.21	0.051
6-10 (Alpha)	Occipital	-0.05	0.681
	Parietal	-0.05	0.678
	Central	0.01	0.948
	Frontal	0.05	0.639
8-12 (Alpha)	Occipital	-0.09	0.434
	Parietal	-0.03	0.789
	Central	0.05	0.666
	Frontal	0.09	0.439
10-15 (Low Beta)	Occipital	-0.11	0.325
	Parietal	0.03	0.802
	Central	0.07	0.506
	Frontal	0.10	0.375
20-30 (High Beta)	Occipital	-0.05	0.676
	Parietal	0.02	0.889
	Central	0.07	0.533
	Frontal	0.10	0.350
12-30 (Beta)	Occipital	-0.07	0.515
	Parietal	0.08	0.472
	Central	0.11	0.331
	Frontal	0.12	0.256
	**Eyes Open**		
2-6 (Theta)	Occipital	0.03	0.758
	Parietal	-0.04	0.703
	Central	0.20	0.064
	Frontal	-0.02	0.834
4-8 (Theta)	Occipital	0.02	0.861
	Parietal	-0.05	0.631
	Central	0.10	0.374
	Frontal	-0.07	0.501
6-10 (Alpha)	Occipital	-0.11	0.304
	Parietal	-0.10	0.386
	Central	0.05	0.641
	Frontal	0.04	0.722
8-12 (Alpha)	Occipital	-0.15	0.165
	Parietal	-0.07	0.520
	Central	0.04	0.739
	Frontal	-0.06	0.578
10-15 (Low Beta)	Occipital	-0.01	0.923
	Parietal	0.02	0.847
	Central	0.25	0.021
	Frontal	0.00	0.973
20-30 (High Beta)	Occipital	-0.07	0.512
	Parietal	-0.04	0.722
	Central	0.07	0.518
	Frontal	0.03	0.799
12-30 (Beta)	Occipital	-0.03	0.757
	Parietal	-0.01	0.908
	Central	0.13	0.229
	Frontal	0.05	0.626

Table S4: Relative Power difference of Pre-medication and Post-medication. (EO/EC)

Band	Lobs	R	P-value
**Pre-medication**			
2-6 (Theta)	Occipital	-0.04	0.722
	Parietal	-0.02	0.883
	Central	0.10	0.389
	Frontal	0.06	0.590
4-8 (Theta)	Occipital	0.12	0.281
	Parietal	0.07	0.529
	Central	0.12	0.279
	Frontal	0.02	0.861
6-10 (Alpha)	Occipital	0.12	0.267
	Parietal	-0.03	0.775
	Central	0.06	0.590
	Frontal	-0.14	0.217
8-12 (Alpha)	Occipital	-0.02	0.862
	Parietal	-0.14	0.207
	Central	-0.12	0.263
	Frontal	-0.22	0.044
10-15 (Low Beta)	Occipital	-0.12	0.285
	Parietal	-0.06	0.580
	Central	-0.15	0.176
	Frontal	-0.17	0.119
20-30 (High Beta)	Occipital	0.09	0.395
	Parietal	0.10	0.382
	Central	-0.14	0.195
	Frontal	-0.08	0.449
12-30 (Beta)	Occipital	0.11	0.305
	Parietal	0.07	0.526
	Central	-0.18	0.093
	Frontal	-0.09	0.434
**Post-medication**			
2-6 (Theta)	Occipital	-0.17	0.118
	Parietal	-0.03	0.817
	Central	-0.11	0.344
	Frontal	-0.17	0.129
4-8 (Theta)	Occipital	0.04	0.709
	Parietal	-0.09	0.411
	Central	0.02	0.826
	Frontal	-0.05	0.630
6-10 (Alpha)	Occipital	0.19	0.078
	Parietal	-0.08	0.477
	Central	0.09	0.415
	Frontal	0.09	0.412
8-12 (Alpha)	Occipital	0.25	0.024
	Parietal	0.06	0.601
	Central	0.14	0.194
	Frontal	0.18	0.107
10-15 (Low Beta)	Occipital	0.23	0.034
	Parietal	0.06	0.605
	Central	0.14	0.188
	Frontal	0.18	0.105
20-30 (High Beta)	Occipital	-0.11	0.317
	Parietal	-0.17	0.127
	Central	-0.13	0.247
	Frontal	-0.06	0.604
12-30 (Beta)	Occipital	0.01	0.936
	Parietal	-0.15	0.176
	Central	-0.05	0.670
	Frontal	-0.01	0.915

Table S5: Relative Power difference of Eyes Close and Eyes Open. (Pre - Post) / Pre

Band	Lobs	R	P-value
**Eyes Close**			
2-6 (Theta)	Occipital	0.07	0.541
	Parietal	0.18	0.108
	Central	0.17	0.131
	Frontal	0.18	0.095
4-8 (Theta)	Occipital	-0.07	0.518
	Parietal	-0.12	0.274
	Central	-0.09	0.420
	Frontal	-0.15	0.192
6-10 (Alpha)	Occipital	-0.12	0.294
	Parietal	-0.06	0.592
	Central	-0.15	0.190
	Frontal	-0.13	0.254
8-12 (Alpha)	Occipital	-0.17	0.134
	Parietal	-0.17	0.123
	Central	-0.10	0.360
	Frontal	-0.02	0.858
10-15 (Low Beta)	Occipital	-0.22	0.047
	Parietal	-0.14	0.211
	Central	-0.09	0.432
	Frontal	-0.09	0.423
20-30 (High Beta)	Occipital	-0.02	0.880
	Parietal	-0.05	0.678
	Central	0.03	0.775
	Frontal	-0.11	0.346
12-30 (Beta)	Occipital	-0.11	0.327
	Parietal	-0.11	0.317
	Central	-0.04	0.693
	Frontal	-0.12	0.279
**Eyes Open**			
2-6 (Theta)	Occipital	0.06	0.577
	Parietal	0.02	0.854
	Central	0.22	0.049
	Frontal	0.10	0.393
4-8 (Theta)	Occipital	0.03	0.811
	Parietal	-0.01	0.923
	Central	0.00	0.977
	Frontal	-0.09	0.403
6-10 (Alpha)	Occipital	-0.16	0.164
	Parietal	-0.13	0.256
	Central	-0.12	0.267
	Frontal	-0.22	0.052
8-12 (Alpha)	Occipital	-0.08	0.484
	Parietal	-0.04	0.694
	Central	-0.08	0.500
	Frontal	-0.18	0.118
10-15 (Low Beta)	Occipital	0.06	0.585
	Parietal	0.01	0.930
	Central	0.01	0.944
	Frontal	-0.09	0.445
20-30 (High Beta)	Occipital	0.06	0.637
	Parietal	0.03	0.797
	Central	-0.02	0.870
	Frontal	-0.08	0.486
12-30 (Beta)	Occipital	-0.01	0.911
	Parietal	0.02	0.832
	Central	0.00	0.978
	Frontal	-0.04	0.791

Table S6: Relative Power difference of Eyes Close and Eyes Open. (Pre / Post)

Band	Lobs	R	P-value
	**Eyes Close**		
2-6 (Theta)	Occipital	0.10	0.361
	Parietal	0.14	0.209
	Central	0.16	0.146
	Frontal	0.15	0.162
4-8 (Theta)	Occipital	0.08	0.492
	Parietal	-0.02	0.828
	Central	0.02	0.879
	Frontal	-0.06	0.561
6-10 (Alpha)	Occipital	-0.03	0.789
	Parietal	-0.12	0.276
	Central	-0.06	0.610
	Frontal	-0.09	0.412
8-12 (Alpha)	Occipital	-0.22	0.044
	Parietal	-0.17	0.119
	Central	-0.06	0.565
	Frontal	0.02	0.872
10-15 (Low Beta)	Occipital	-0.20	0.075
	Parietal	-0.18	0.107
	Central	-0.03	0.840
	Frontal	-0.01	0.921
20-30 (High Beta)	Occipital	-0.02	0.869
	Parietal	-0.07	0.546
	Central	0.00	0.986
	Frontal	-0.01	0.962
12-30 (Beta)	Occipital	-0.01	0.962
	Parietal	-0.07	0.532
	Central	-0.02	0.886
	Frontal	-0.03	0.770
	**Eyes Open**		
2-6 (Theta)	Occipital	-0.03	0.803
	Parietal	0.14	0.200
	Central	0.21	0.051
	Frontal	0.20	0.074
4-8 (Theta)	Occipital	0.00	0.995
	Parietal	0.04	0.746
	Central	0.03	0.769
	Frontal	-0.07	0.536
6-10 (Alpha)	Occipital	-0.06	0.591
	Parietal	-0.09	0.394
	Central	-0.07	0.499
	Frontal	-0.12	0.284
8-12 (Alpha)	Occipital	-0.05	0.625
	Parietal	-0.14	0.204
	Central	-0.11	0.310
	Frontal	-0.16	0.134
10-15 (Low Beta)	Occipital	0.05	0.633
	Parietal	-0.07	0.545
	Central	0.02	0.889
	Frontal	0.12	0.274
20-30 (High Beta)	Occipital	-0.06	0.595
	Parietal	-0.16	0.148
	Central	-0.12	0.290
	Frontal	0.01	0.929
12-30 (Beta)	Occipital	-0.05	0.628
	Parietal	-0.14	0.215
	Central	-0.09	0.406
	Frontal	-0.04	0.743

Table S7: Relative Power EC of Pre-medication and Post-medication

Band	Lobs	R	P-value
**Pre-medication**			
2-6 (Theta)	Occipital	0.03	0.819
	Parietal	-0.05	0.646
	Central	-0.14	0.218
	Frontal	-0.14	0.198
4-8 (Theta)	Occipital	0.02	0.853
	Parietal	-0.08	0.451
	Central	0.05	0.666
	Frontal	0.13	0.224
6-10 (Alpha)	Occipital	0.05	0.666
	Parietal	-0.06	0.560
	Central	0.12	0.275
	Frontal	0.08	0.463
8-12 (Alpha)	Occipital	0.09	0.399
	Parietal	-0.03	0.756
	Central	0.08	0.469
	Frontal	0.17	0.119
10-15 (Low Beta)	Occipital	-0.02	0.878
	Parietal	-0.08	0.462
	Central	0.05	0.624
	Frontal	0.05	0.637
20-30 (High Beta)	Occipital	-0.08	0.491
	Parietal	-0.06	0.619
	Central	0.12	0.295
	Frontal	-0.02	0.844
12-30 (Beta)	Occipital	-0.14	0.199
	Parietal	-0.07	0.532
	Central	0.05	0.682
	Frontal	-0.03	0.803
**Post-medication**			
2-6 (Theta)	Occipital	0.18	0.098
	Parietal	0.11	0.300
	Central	-0.06	0.587
	Frontal	0.05	0.679
4-8 (Theta)	Occipital	0.04	0.726
	Parietal	-0.01	0.894
	Central	-0.01	0.948
	Frontal	-0.01	0.930
6-10 (Alpha)	Occipital	-0.07	0.553
	Parietal	0.08	0.482
	Central	0.21	0.059
	Frontal	0.13	0.223
8-12 (Alpha)	Occipital	0.02	0.889
	Parietal	0.07	0.559
	Central	0.20	0.069
	Frontal	0.04	0.747
10-15 (Low Beta)	Occipital	0.02	0.880
	Parietal	0.11	0.301
	Central	0.08	0.488
	Frontal	-0.01	0.892
20-30 (High Beta)	Occipital	-0.01	0.476
	Parietal	-0.06	0.587
	Central	-0.01	0.917
	Frontal	-0.08	0.455
12-30 (Beta)	Occipital	-0.14	0.199
	Parietal	-0.05	0.633
	Central	0.02	0.886
	Frontal	-0.09	0.395

Table S8: Relative Power EO of Pre-medication and Post-medication

Band	Lobs	R	P-value
**Pre-medication**			
2-6 (Theta)	Occipital	-0.04	0.715
	Parietal	-0.09	0.430
	Central	-0.01	0.901
	Frontal	0.02	0.842
4-8 (Theta)	Occipital	0.21	0.053
	Parietal	0.09	0.424
	Central	0.23	0.034
	Frontal	0.29	0.009
6-10 (Alpha)	Occipital	0.00	0.989
	Parietal	-0.03	0.802
	Central	0.03	0.776
	Frontal	0.12	0.292
8-12 (Alpha)	Occipital	-0.11	0.329
	Parietal	-0.18	0.098
	Central	-0.13	0.231
	Frontal	-0.14	0.198
10-15 (Low Beta)	Occipital	-0.07	0.507
	Parietal	-0.22	0.040
	Central	-0.21	0.051
	Frontal	-0.22	0.047
20-30 (High Beta)	Occipital	0.19	0.087
	Parietal	0.02	0.835
	Central	-0.02	0.870
	Frontal	-0.06	0.592
12-30 (Beta)	Occipital	0.19	0.084
	Parietal	0.10	0.349
	Central	-0.07	0.498
	Frontal	-0.09	0.396
**Post-medication**			
2-6 (Theta)	Occipital	-0.03	0.795
	Parietal	-0.03	0.785
	Central	-0.23	0.033
	Frontal	-0.19	0.086
4-8 (Theta)	Occipital	0.01	0.946
	Parietal	-0.03	0.816
	Central	0.06	0.556
	Frontal	0.09	0.410
6-10 (Alpha)	Occipital	0.16	0.145
	Parietal	0.19	0.088
	Central	0.37	0.001
	Frontal	0.26	0.016
8-12 (Alpha)	Occipital	0.24	0.027
	Parietal	0.26	0.019
	Central	0.41	0.000140
	Frontal	0.27	0.013
10-15 (Low Beta)	Occipital	0.29	0.008
	Parietal	0.31	0.003
	Central	0.38	0.000335
	Frontal	0.36	0.001
20-30 (High Beta)	Occipital	-0.26	0.016
	Parietal	-0.17	0.128
	Central	0.03	0.814
	Frontal	0.00	0.976
12-30 (Beta)	Occipital	-0.17	0.127
	Parietal	-0.13	0.249
	Central	0.09	0.399
	Frontal	0.10	0.366
